# Oncolytic reovirus: tumor-microenvironment remodeling, engineering constraints, and translational strategies

**DOI:** 10.3389/fimmu.2026.1956202

**Published:** 2026-09-28

**Authors:** Rui Li, Xiafei Liu, Xiliang Cong, Sisi Dou, Qing Wang, Bingjie Lv, Weihong Ren

**Affiliations:** 1 Department of Laboratory Medicine, The First Affiliated Hospital of Henan University of Chinese Medicine, Zhengzhou, China; 2 The First Clinical Medical College of Henan University of Chinese Medicine, Zhengzhou, China; 3 Zhengzhou University Affiliated Cancer Hospital, Zhengzhou, China

**Keywords:** oncolytic reovirus, tumor microenvironment, viral engineering, combination therapy, delivery systems

## Abstract

Oncolytic reovirus (ReoV) has emerged as a promising cancer immunotherapy platform because of its natural tumor tropism and immunostimulatory properties. Reovirus enters susceptible cells through receptors including junctional adhesion molecule-A and exploits oncogenic signaling and impaired antiviral responses to preferentially replicate in tumor cells. Viral replication induces apoptosis and other context-dependent forms of programmed cell death, while tumor lysis promotes antigen release and immune activation. However, reovirus monotherapy has shown limited clinical efficacy because of circulating neutralizing antibodies, heterogeneous receptor expression, stromal barriers, and the immunosuppressive tumor microenvironment. Current strategies therefore combine reovirus with chemotherapy, radiotherapy, immune checkpoint blockade, and targeted agents, although treatment outcomes remain dependent on tumor type, dose, and scheduling. Unlike previous reviews focused primarily on reovirus biology or individual combination regimens, this review examines how reovirus remodels the tumor microenvironment and critically assesses the translational limitations of combination therapy. We further discuss reverse-genetics platforms, capsid and receptor-targeting modifications, immunomodulatory transgenes, and carrier-based delivery systems. Future development will require biomarker-guided patient selection, genetically stable viral constructs, optimized systemic delivery, and prospective evidence that immune activation produces clinically meaningful benefit.

## 1 Introduction

Current treatments for malignant tumors include surgery, radiotherapy, chemotherapy, targeted therapy, and endocrine therapy (1). Although these approaches can produce initial tumor responses, limited tumor selectivity, systemic toxicities, and acquired resistance often restrict long-term clinical benefit (2). In response to this clinical need, oncolytic virus (OV) therapy has emerged as a therapeutic strategy. Its antitumor efficacy stems from a unique dual mechanism: it not only selectively replicates in tumor cells and induces *in situ* lysis but also releases tumor antigens and generates “*in situ* vaccine” effect. This process can reverse the local immunosuppressive state and drive a broad and sustained systemic antitumor immune response. Among various viral vectors, reovirus has garnered significant attention due to its unique biological properties (3). First, ReoV facilitates endocytosis by binding to the highly expressed JAM-A receptor on the surface of tumor cells. Second, it exploits aberrant activation of the RAS signaling pathway, which is commonly found in many solid tumors, to achieve specific and efficient replication within the cells. Ultimately, ReoV kills infected tumor cells through mechanisms such as inducing apoptosis, while generally showing limited pathogenicity in immunocompetent hosts (4). However, as multiple early clinical trials have progressed, the limitations of relying solely on ReoV monotherapy have become apparent. Neutralizing antibodies in the bloodstream, dense extracellular matrix barriers of tumor cells, and complex immunosuppressive microenvironments often significantly reduce the efficiency of targeted viral delivery and sustained cytotoxicity (5).To overcome these translational barriers, current research and development efforts have shifted towards the comprehensive integration of multidimensional strategies. On the one hand, there is a focus on combining existing methods such as chemotherapy, radiotherapy, and immune checkpoint inhibitors to achieve synergistic effects and reverse resistance. On the other hand, molecular modifications of the virus are being pursued through genetic engineering techniques, including reverse genetics, to modify capsid proteins and encode immunomodulatory payloads, or develop novel delivery systems. This paper thoroughly explores the biological characteristics of reovirus, its oncolytic mechanisms, and the latest advancements in gene modification, combination strategies, and delivery systems. The aim is to provide insights for the future development of a new generation of efficient and precise cancer therapies.

## 2 Biological characteristics of ReoV

### 2.1 Molecular architecture and clinical translation of oncolytic reovirus

Reovirus is a non-enveloped, icosahedral virus approximately 85 nm in diameter. Its segmented double-stranded RNA genome is encapsulated by a concentric protein shell composed of an outer capsid and a core (6). Each genomic segment encodes a specific viral protein. Among these, the three larger segments (L1, L2, and L3) express the λ3, λ2, and λ1 proteins, respectively. The λ3 protein possesses RNA-dependent RNA polymerase (RdRp) activity, while λ2 functions as both a capping enzyme and a core structural protein. Additionally, λ1 contributes to the composition of the core structure. The three medium-sized segments (M1, M2, and M3) encode the μ1, μ2, and μNS proteins, with μ1 being essential for viral membrane penetration, and μ2 and μNS serving as critical non-structural proteins that facilitate the formation of viral replication factories. Additionally, the four smaller segments (S1, S2, S3, and S4) encode proteins σ1, σ2, σ3, and σNS. The σ1 protein functions as a cell adhesion protein and can produce its smaller variant, σ1s, through an alternative translation initiation mechanism. Collectively, these structural and nonstructural proteins support viral entry, genome replication, virion assembly, and interactions with host-cell pathways (7). Together, these components drive the infection cycle, which includes attachment, entry, replication, and assembly phases (8). This cycle serves as the prerequisite and foundation for viral oncolysis. To date, three species of orthoreovirus, including mammalian orthoreovirus (MRV), avian orthoreovirus (ARV), and pteropine orthoreovirus (PRV), have demonstrated oncolytic activity. Pelareorep, a clinical-grade isolate derived from mammalian orthoreovirus type 3 Dearing, registered as Reolysin^®^, has been evaluated in multiple phase I and phase II clinical trials, with a generally manageable safety profile but modest single-agent activity (9).

### 2.2 Determinants of reovirus tropism and replication selectivity

Reovirus exhibits preferential replication in selected tumor cells. Viral entry into susceptible cells occurs through receptor-mediated endocytosis after binding to JAM-A. JAM-A is overexpressed in various cancers, including breast cancer, non-small cell lung cancer, diffuse large B-cell lymphoma, and multiple myeloma (10). Following infection, the outer capsid is proteolytically disassembled within acidified endosomes, leading to the release of the transcriptionally active core. The transcription and translation events necessary for progeny virus assembly occur within viral inclusions located in the cytoplasm (11).The reovirus life cycle occurs entirely in the cytoplasm and does not require entry into the host-cell nucleus (10). Reovirus replication is also influenced by oncogenic RAS signaling and defects in host antiviral responses. In cells with intact antiviral signaling, the virus’s double-stranded RNA (dsRNA) activates the host cell’s double-stranded RNA-dependent protein kinase (PKR), leading to the phosphorylation of eukaryotic initiation factor 2α (eIF2α) and subsequently inhibiting viral protein synthesis (12). However, in tumor cells with aberrant activation of the RAS signaling pathway, this inhibitory mechanism is disrupted, impairing PKR function and allowing for continued viral replication, ultimately resulting in cell lysis. RAS mutations activate the downstream phosphoinositide 3-kinase (PI3K)/mitogen-activated protein kinase (MAPK)/extracellular signal–regulated kinase (ERK) signaling axis and inhibit translation of the RIG-I–encoding Ddx58 mRNA, which reduces expression of this pattern-recognition receptor and weakens cellular surveillance of viral RNA (13). The virus-encoded σ3 protein sequesters viral dsRNA and prevents recognition by PKR, allowing the virus to evade innate immune detection (14). Concurrently, RAS activation upregulates cathepsin B/L activity and promotes viral particle uncoating, which increases infection efficiency (15). In addition, RAS is activated in about 30% of malignancies, and aberrant pathways such as PI3K–Akt, mTOR, and NF-κB in some tumors create an intracellular environment that favors viral replication (16). Overall, metabolic reprogramming that accompanies tumor transformation and defects in immune surveillance together provide the essential basis for ReoV’s selective replication. Activation of RAS signaling promotes maturation of intermediate subviral particles (ISVPs) and enhances the virus’s cytopathic effects; these factors may collectively contribute to the preferential replication and cytotoxicity of ReoV in selected tumor cells (**Figure 1**).

**Figure 1:**
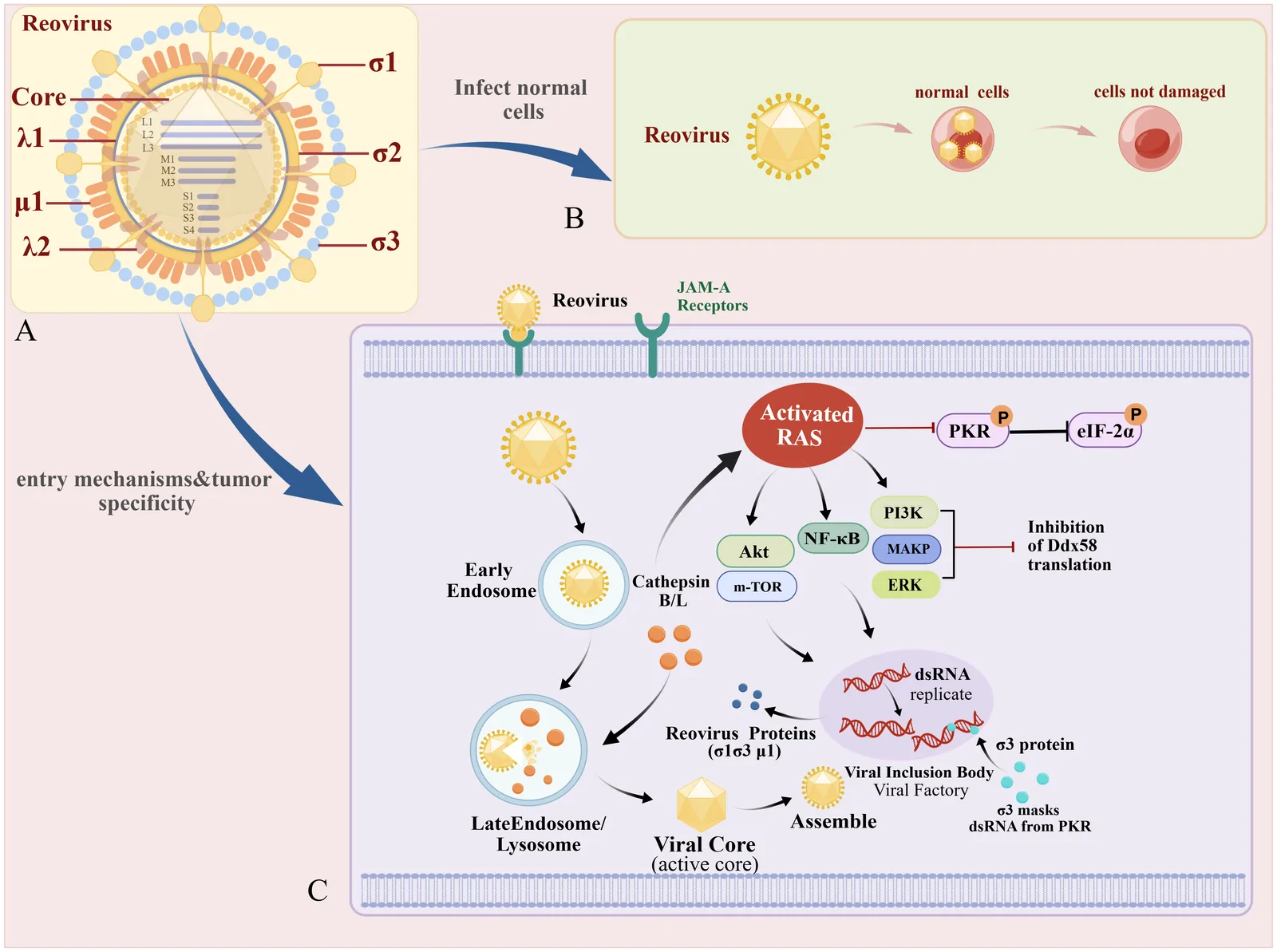
**(A)** Shows the internal structure of reovirus. **(B)** Shows reovirus infecting a normal cell, and **(C)** illustrates the specific binding of the reovirus to junction adhesion molecule A (JAM-A). This interaction facilitates viral entry into susceptible cells via receptor-mediated endocytosis. In addition, the figure shows the regulation of RAS pathway activation. Solid black arrows indicate direct biological processes or activation, red blunt-tipped lines indicate inhibition or impairment; black blunt-tipped lines indicate the induction of phosphorylation; colored arrows indicate normal biological responses. Created with BioGDP.com.

## 3 Mechanism of oncolytic action of ReoV

The direct lytic cytotoxicity of ReoV represents its most significant oncolytic effect. This virus exploits oncogenic alterations and impaired antiviral signaling in tumor cells, allowing it to replicate extensively within these cells. This replication can induce apoptosis and other context-dependent cell-death pathways, ultimately leading to the lysis of tumor cells (17). However, the antitumor potential of ReoV extends beyond direct cell lysis. In addition to causing the destruction of tumor cells, the localized replication of the virus can elicit robust local and systemic immune responses, effectively overcoming local immunosuppression within the tumor microenvironment. In certain instances, this remodeling of the immune microenvironment may prove more critical than the virus’s direct oncolytic action (18).

### 3.1 Direct oncolytic effects of ReoV

#### 3.1.1 ReoV-induced programmed cell death

Reovirus-mediated tumor cell lysis frequently involves activation of apoptotic signaling. After infection, cytoplasmic double-stranded RNA is detected by pattern-recognition receptors such as PKR, retinoic acid-induced gene I (RIG-I), or melanoma differentiation–associated protein 5 (MDA5). Intracellular signaling can also be initiated through σ1- and μ1-mediated receptor binding and membrane penetration. These signals typically trigger interferon (IFN) production and activation of nuclear factor κB (NF-κB). Activation of NF-κB and IRF3 promotes the expression of inflammatory cytokines and death-inducing ligands, including tumor necrosis factor–related apoptosis-inducing ligand (TRAIL). TRAIL subsequently binds to cell-surface death receptors and activates caspase-dependent apoptosis. TRAIL then binds to cell-surface death receptors, activates caspase-3 and caspase-7, and ultimately induces apoptosis. Although interferons (IFNs) play a key role in promoting cell death, they are not strictly required for apoptosis triggered by reovirus; this helps explain why tumor cells lacking an IFN response can still undergo virus-induced apoptosis (19). Inhibiting apoptotic caspases does not always abolish reovirus-induced cell death, indicating that other forms of programmed cell death (PCD) can occur (20). Additional forms of programmed cell death have been proposed in selected models; one such pathway, necroptosis, is a caspase-independent form of PCD that depends on the receptor-interacting serine/threonine-protein kinase 3 (RIPK3) and its substrate, the pseudokinase mixed lineage kinase domain-like protein (MLKL) (21). Necroptosis is highly inflammatory, and reovirus-induced necroptosis may enhance the oncolytic potential of this virus. This enhancement occurs not only through the promotion of necrotic cell lysis in infected cells but also by modulating the inflammatory tumor microenvironment and stimulating beneficial antitumor immune responses (22). Simultaneously, studies have indicated that reovirus can disrupt the protective autophagy pathway in normal cells, which not only facilitates its own replication but also enhances the death of tumor cells (23).

### 3.2 Mechanisms by which ReoV remodels the tumor microenvironment

#### 3.2.1 Activation of immune cells

Tumors evade immune-mediated clearance through impaired antigen recognition, insufficient lymphocyte activation, and restricted lymphocyte trafficking (24). Reovirus selectively infects and lyses tumor cells, releasing tumor-associated antigens (TAAs) into the tumor microenvironment and triggering dendritic cell (DC) maturation. Reovirus also enhances the ability of antigen-presenting cells (APCs) to process tumor antigens and present them to CD8^+^ T cells, thereby promoting CD8^+^ T-cell activation, increasing the release of proinflammatory cytokines and chemokines, and strengthening antitumor immunity (25).Preclinical studies (26) showed that intravenous administration of reovirus reduced lymph-node metastasis in a B16 melanoma model, while eliciting immunostimulatory cytokines and inducing CD8^+^ T cell responses to endogenous tumor-associated antigens. When human myeloid dendritic cells (DCs) were cocultured with reovirus-infected melanoma cells, the DCs secreted cytokines associated with natural killer (NK) cell activation, particularly interleukin-12 (IL-12). Upon physical contact between the two cell types, reovirus-loaded DCs induced NK cells to produce interferon-γ (IFN-γ), and the cytokines released by those DCs promoted NK cell migration. Data indicate that dendritic cell (DC) loading with reovirus enhances recruitment of immune cells to primary and secondary tumor microenvironments, which helps reduce tumor survival (27).

#### 3.2.2 Reversal of immunosuppression

The tumor microenvironment is typically enriched with immunosuppressive populations such as myeloid-derived suppressor cells (MDSCs), tumor-associated macrophages (TAMs), and CD4+FoxP3+ regulatory T cells (Tregs). These cells release inhibitory cytokines, including interleukin-10 (IL-10) and transforming growth factor-β1 (TGF-β1), which suppress the maturation and function of effector T cells, natural killer (NK) cells, and dendritic cells (DCs) and thereby weaken antigen recognition and presentation by these effector cells (28). Studies have shown that blocking MDSC recruitment via a C-C chemokine receptor 2 (CCR2)-dependent mechanism or by using gemcitabine enhanced the antitumor effects induced by reovirus (29). Katayama et al (30) found that in lymphoma- and melanoma-bearing mice, following internalization by MDSCs, the ReoV dsRNA genome is exposed to Toll-like receptor 3 (TLR3), which then suppresses the immunosuppressive activity of MDSCs. Overcoming the immunosuppressive environment within tumor-bearing hosts is therefore essential to effectively activate antitumor immune responses. Although no definitive studies have yet elucidated how reovirus interacts with tumor-associated macrophages (TAMs) and regulatory T cells (Tregs), evidence from related oncolytic-virus models provides indirect support for targeting TAMs and Tregs in ReoV-based therapy. For example, myeloid cells have been shown to drive Treg differentiation in the tumor microenvironment through direct cell–cell interactions (31). In multiple models, including Ewing sarcoma and glioblastoma, depletion of TAMs markedly improved the antitumor efficacy of oncolytic herpes simplex virus (HSV) by substantially altering the tumor microenvironment (32). These findings therefore support the future application of TAM- and Treg-targeting strategies in ReoV-based cancer immunotherapy.

#### 3.2.3 Killing tumor-associated fibroblasts

Cancer-associated fibroblasts (CAFs) are major regulators of the tumor microenvironment; they shape conditions that favor tumor growth through immune suppression, formation of physical barriers, and promotion of tumor progression (33, 34). Nozomi Kurisu et al (35)found that ReoV was noncytotoxic to mouse tail-tip fibroblasts but was cytotoxic to primary CAFs isolated from mouse subcutaneous tumors. ReoV entered and infected mouse primary CAFs through a JAM-A- and cathepsin L-dependent pathway, then activated the RIG-I/IPS-1 innate immune signaling cascade, which led to robust production of IFN-β and ISGs. This signaling further induced expression of key proapoptotic genes such as TRAIL, Noxa, and PUMA, and ultimately activated caspase-3, culminating in apoptosis, thereby effectively killing CAFs.

#### 3.2.4 Anti-angiogenesis

Rapidly growing tumors can outstrip their vascular supply, creating regions of hypoxia and nutrient deprivation. Under these conditions, tumor cells secrete abundant pro-angiogenic factors and cytokines that disrupt local homeostasis and stimulate neovascularization. This increase in proangiogenic signaling promotes abnormal endothelial sprouting, producing chaotic, immature tumor vessels that differ morphologically and functionally from normal vasculature; they are tortuous and leaky, with unstable endothelium and sparse or absent pericyte coverage. During this process, vascular dysfunction establishes a self-reinforcing cycle of hypoxia and angiogenesis. Under severe hypoxia, the hypoxic tumor microenvironment can impair treatment efficacy by reducing delivery of anticancer drugs, diminishing leukocyte function, and causing inefficient immune cell trafficking, all of which are critical for immunotherapy (36, 37). Jennifer S, Carew et al. found that reovirus suppresses angiogenesis in both wild-type and RAS-mutant soft-tissue sarcoma cells through three mechanisms (38). First, ReoV strongly upregulated and promoted secretion of interferon-γ–induced protein 10 (IP-10/CXCL10), thereby blocking vascular endothelial growth factor (VEGF)–induced endothelial tube formation. Second, ReoV markedly reduced the activity of hypoxia-inducible factor 1α (HIF-1α). This inhibition directly caused a pronounced decrease in VEGF secretion and thereby suppressed angiogenesis. Finally, compared with monotherapy, combining ReoV with targeted agents further increased CXCL10 expression and more strongly inhibited angiogenesis, with the temsirolimus combination producing the strongest effect.

#### 3.2.5 Metabolic constraints on ReoV replication

Tumorigenesis is closely linked to cellular metabolic reprogramming, a process driven directly or indirectly by oncogenic mutations. Tumor cells undergo metabolic adaptation that supports survival and biomass production under nutrient-limited conditions. Metabolic remodeling associated with tumors alters metabolite levels both intracellularly and in the extracellular milieu, and these changes influence gene expression patterns, cell differentiation states, and features of the tumor microenvironment (39). Kennedy et al (40) found that ReoV infection induces oxidative stress in cancer cells, which leads to inhibitory phosphorylation of PDH and blocks pyruvate entry into the TCA cycle, creating a metabolic state that is unfavorable for viral replication. Pharmacological inhibition or genetic depletion of PDK1 restored PDH activity and enhanced ReoV oncolysis *in vitro* and *in vivo* (**Figure 2**).

**Figure 2:**
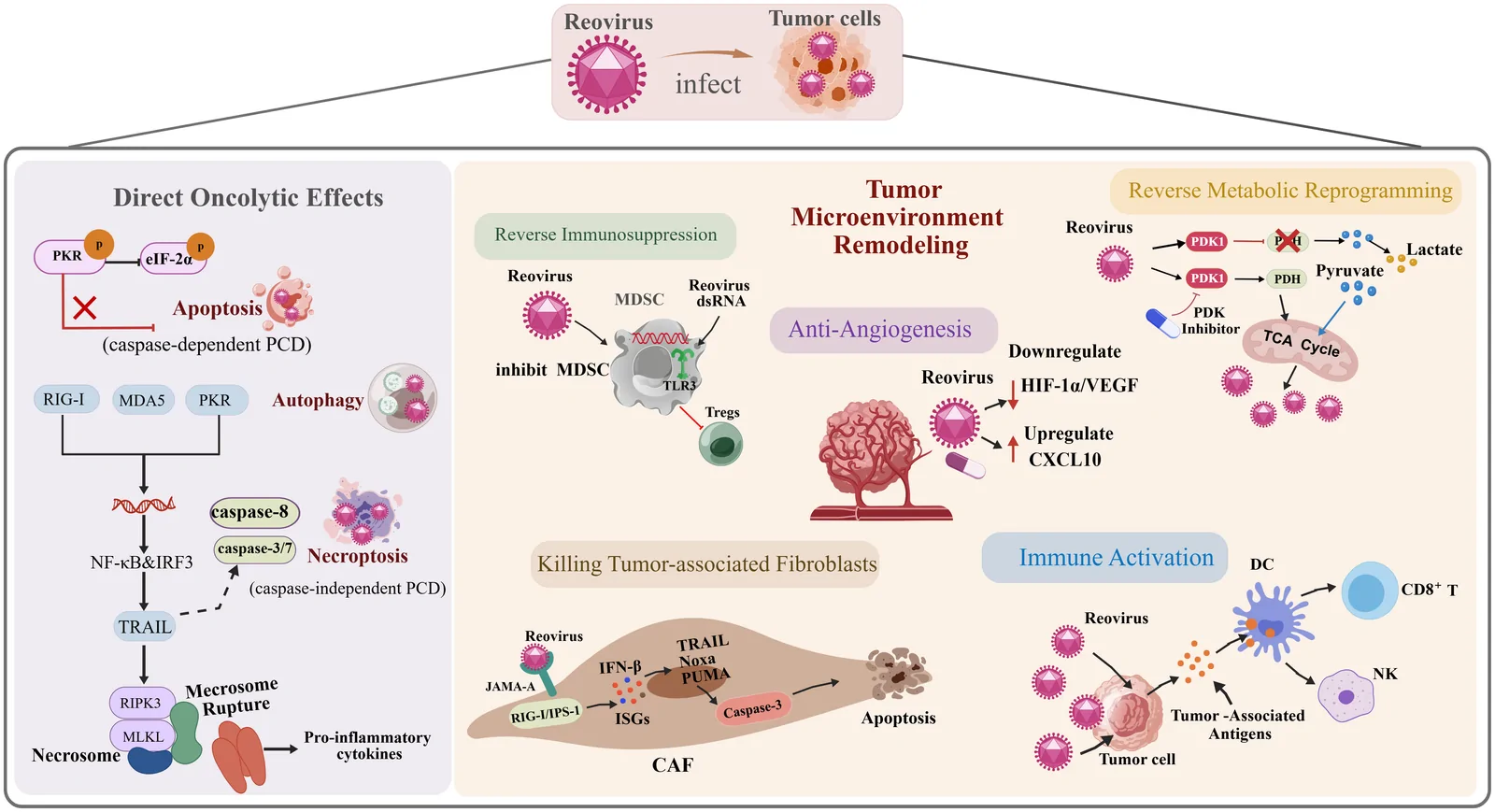
There are two mechanisms by which reovirus infects tumor cells: one is a direct oncolytic mechanism, which includes apoptosis, programmed cell death, and autophagy; the other is an oncolytic effect achieved by reshaping the tumor microenvironment. Proposed mechanisms by which ReoV remodels the tumor microenvironment, including immune-cell activation, modulation of immunosuppressive myeloid cells, targeting of cancer-associated fibroblasts, inhibition of angiogenic signaling, and alteration of tumor-cell metabolism. Red blunt-tipped lines indicate inhibition; a solid red arrow indicates an increase or decrease solid; black arrows indicate direct effects; dashed arrows indicate transcriptional regulation. Created with BioGDP.com.

## 4 Engineering strategies for ReoV

Although wild-type strains have shown favorable oncolytic properties in preclinical and clinical studies, researchers still face the challenge of minimizing antiviral immunity while maximizing antitumor efficacy. ReoV engineering strategies are being developed to improve tumor targeting, enhance intratumoral spread, and modulate antitumor immunity.

### 4.1 Reverse genetics systems and modification platforms

Early studies established reverse-genetics systems for the generation of recombinant reovirus expressing exogenous genes. This technology not only serves as a powerful tool for fundamental research on double-stranded RNA viruses but also presents a promising translational platform for the future development of safe and effective oncolytic therapies and gene therapies (41). Subsequently, other researchers applied this reverse genetics system to investigate two prototype viral strains, T1L and T3D. By integrating cDNA fragments from multiple genes onto a single plasmid—reducing the number of plasmids from 10 in the first generation to 4 in the second—increased viral rescue efficiency. By combining multiple cDNA fragments into four plasmids rather than the original ten-plasmid system, the revised platform improved viral rescue efficiency. The use of BHK cells expressing T7 RNA polymerase further shortened the rescue process (42).

### 4.2 Engineering of viral mutants and enhancement of oncolytic activity

A study demonstrated that recombinant viruses produced in triple-negative breast cancer cells through forward genetics exhibit greater infectivity in MDA-MB-231 breast cancer cells than the parental ReoV. When combined with topoisomerase inhibitors identified via high-throughput screening, the infectivity and cytotoxicity of the recombinant ReoV against triple-negative breast cancer cells were further increased, supporting further evaluation of this approach in triple-negative breast cancer (TNBC) models with this cancer subtype (43). Additionally, a genetically engineered reovirus mutant, designated SV5, was developed; this mutant enhanced viral spread and oncolytic activity by reducing attachment to tumor cells, thereby exhibiting significantly improved tumor-suppressing effects compared to the wild-type T3D in a HER2-positive breast cancer mouse model (44). To tackle the suboptimal efficacy of immunotherapy in treating prostate cancer (45), especially castration-resistant forms, jin-3 infected prostate cancer cells through a partially JAM-A-independent mechanism. Jin-3 showed oncolytic activity and the capacity to trigger immunogenic cell death in diverse preclinical models, including PDX models. These findings not only suggest jin-3 as a potential candidate for prostate cancer immunotherapy but also underscore the potential translational relevance of patient-derived tumor models in assessing treatment efficacy and informing the preclinical evaluation of oncolytic virus sensitivity. Investigation into the oncolytic properties of reoviruses in bladder cancer immunotherapy revealed that jin-3 notably stimulates markers of immunogenic cell death, interferon-stimulated genes, and inflammatory molecules, demonstrating more potent immunomodulatory effects compared to the wild-type strain R124 (46). Jin-3 has shown superior efficacy in a dose-dependent manner by significantly increasing the expression of immune cell death markers, interferon-stimulated genes, and inflammatory cytokines. In a co-culture system involving tumor cells and peripheral blood mononuclear cells, the infection of bladder tumor organoids with jin-3 resulted in enhanced cancer cell death when co-cultured with immune cells. This enhancement was accompanied by the production of such as CXCL10 and IFN-γ. In conclusion, the jin-3 mutant elicits a more robust immune-stimulatory response and holds promise as a potential candidate for clinical translation in urothelial carcinoma of the bladder (UCB) (47).

### 4.3 σ1 engineering and receptor retargeting

Researchers (48) applied reverse genetics to introduce site-specific cysteine modifications at the N-terminal, middle, and C-terminal regions of the σ1 protein. This strategy sought to exploit disulfide-bond cross-linking to reduce conformational flexibility, leading to the creation of diverse recombinant strains. Investigations have revealed that these alterations not only increase viral binding affinity for the JAM-A receptor, enhancing its capacity to improve attachment to and infection of susceptible cells, but also indicate that these modified viruses could demonstrate improved replication efficiency and cell-to-cell spread. Studies have shown that mouse E0771 mammary carcinoma cells exhibit significant resistance to reovirus T3DPL because of low JAM-A expression. By altering the head domain of the reovirus σ1 protein, for example, by incorporating an RGD motif or introducing mutations in the sialic acid-binding domain, the virus can circumvent the limitations imposed by the high-affinity receptor (JAM-A) at 37 °C, enabling infection of breast cancer cells with reduced receptor levels. This approach provides a potential strategy for overcoming receptor heterogeneity (49). A T3D variant, termed RP116, was derived from cancer cells with persistent infection, harboring a premature termination codon mutation in the S1 gene. This alteration results in a truncated σ1 attachment protein lacking a globular head. Molecular characterization and evaluation of its antitumor efficacy revealed that RP116 exhibits robust replication in various cancer cell lines while showing decreased reliance on the JAM-A receptor. In syngeneic mouse models, RP116 demonstrated significant inhibition of tumor growth following intratumoral or intravenous administration. Furthermore, when combined with checkpoint inhibitors, RP116 facilitated long-term remission and immune memory. Importantly, RP116 elicited lower titers of T3D-neutralizing antibodies thereby preserving the efficacy of subsequent T3D retreatment (**Table 1**) (50).

**Table 1:** Genetic engineering strategies for ReoV.

Engineering target	Modification	Expected advantage	Evidence model	Main result	Safety/stability concern
MRV reverse genetics platform	Combining cDNA from multiple gene fragments onto a single plasmid	Improve virus recovery efficiency and reduce recovery time	Baby hamster kidney (BHK) cells expressing T7 RNA polymerase	An efficient reverse-genetics system was established	Safe, non-pathogenic vector for gene therapy
Reovirus (mutant associated with TNBC)	Forward-genetics recombinant viruses + HTS-identified topoisomerase inhibitors	Enhanced anti-TNBC infectivity and cytotoxicity	MDA-MB-231 triple-negative breast cancer cells	Higher infectivity (vs. parental ReoV); enhanced cytotoxicity + inhibitor	Not reported
Reovirus mutant (SV5)	Engineered SV5 variant with 5 mutations	Reduced tumor attachment, promoting intratumoral spread and oncolytic efficacy	HER2-positive breast cancer mouse model	Superior anti-tumor efficacy vs WT T3D	Not reported
Reovirus mutant (Jin-3)	Selection and characterization of the jin-3 mutant	Enhancement of oncolytic and immunostimulatory activity	PCa models (incl. PDX), bladder tumor organoids & PBMC co-cultures	JAM-A-independent cell entry Potent induction of ICD and immune mediators (CXCL10, IFN-γ)	Not reported
σ1 attachment protein	Site-specific disulfide cross-linking of Cys residues across head, body, and tail domains	Restricted conformational flexibility → enhanced JAM-A affinity, replication & viral spread		Enhanced precision targeting and infectivity	Not reported
σ1 protein head domain	RGD insertion or sialic acid-binding domain mutation	Decreased JAM-A reliance and enhanced receptor-low cell infectivity	E0771 mouse breast cancer cells that are highly resistant to T3DPL	Efficient infection of receptor-low breast cancer cells at 37 °C	Not reported
σ1 attachment protein	S1 premature stop codon → truncated σ1 lacking globular head	↓JAM-A dependence; enables sequential virotherapy	Immunocompetent syngeneic mouse models	Efficient replication; synergizes with ICB to elicit durable remission and immune memory	Favorable safety,↓NAb induction preserves efficacy of T3D retreatment
S1 genome segment	Reverse genetics-mediated GM-CSF insertion into ReoV S1 segment	↑DC expansion & differentiation→T cell activation →potent antitumor immunity	Pancreatic cancer mouse model	*In vitro*: stable GM-CSF secretion; *In vivo*: systemic DC & T cell activation	Stable secretion of exogenous proteins
S2 fragment (σ2 gene)	Using a 2A peptide linker, fuse the phototoxic fluorescent protein KRmem to the 3’ end of the σ2 gene	Synergistic OV–PDT combination strategy	Gastric cancer cell lines	Functional KRmem mediates photo-induced cytotoxicity in MKN45P cells	Maintained replication kinetics
Avian Reovirus (ARV)	Identification of the Plg-RKT receptor and the mechanism by which p17 regulates Hsp90/Cdc37	Molecular targets for optimizing ARV oncolytic selectivity and replication	B16-F10 mouse melanoma cells	Elucidated the Plg-RKT–Src/p38 MAPK receptor axis and p17-driven viral assembly	Not reported

### 4.4 Recombinant ReoV encoding exogenous genes

A reverse-genetics study (51) generated a recombinant ReoV that expresses mouse or human GM-CSF by inserting the GM-CSF gene into the ReoV S1 genome segment through reverse genetics. This modified virus stably expressed and secreted GM-CSF *in vitro*, stimulating the growth and maturation of dendritic cells (DCs). When administered via intratumoral injection in a pancreatic cancer mouse model, this virus significantly increased systemic DC and T-cell activation. These findings suggest that GM-CSF-expressing ReoV has the potential to enhance antitumor immune responses. To explore the combination of oncolytic virotherapy and photodynamic therapy, a recombinant mammalian orthoreovirus (MRV T3D Dearing strain) was engineered to express the membrane-targeted phototoxic fluorescent protein KillerRed (KRmem) (52). This was accomplished by genetically linking KRmem to the 3’ end of the σ2 viral gene within the S2 segment, employing a 2A peptide linker to enable the expression of multiple proteins from a single transcript. Validation through RNA electrophoresis, Western blot analysis, and immunofluorescence techniques confirmed the successful and functional incorporation of KRmem into the recombinant virus. Additionally, assessments indicated that the replication kinetics of the recombinant virus in cultured cells was comparable to that of the wild-type MRV. Importantly, experimental results demonstrated the infectivity of this recombinant virus in two gastric cancer cell lines, MKN45P and MKN7. Notably, after illumination of infected cells, MKN45P cells exhibited significant cytotoxic effects. These findings highlight the potential of the KRmem-expressing recombinant MRV as a promising therapeutic strategy for gastric cancer.

### 4.5 Comparative engineering insights from avian orthoreovirus

Because avian and mammalian orthoreoviruses differ in host range, receptor usage, and viral proteins, findings from ARV should be regarded as comparative engineering insights rather than direct evidence for pelareorep development. Functional experiments involving mouse melanoma B16-F10 cells identified Plg-RKT as the primary receptor for ARV infection in these cells. Binding of ARV to Plg-RKT activated Src and p38 MAPK signaling, identifying a potential engineering target to enhance the oncolytic selectivity and therapeutic potential of ARV (53). Studies have shown that p17 controls the Hsp90/Cdc37 complex formation, leading to p17 stabilization and facilitating viral protein aggregation and assembly within the viral factory. This mechanism elucidates how ARV exploits host-partner interactions to enhance replication efficiency. These findings identify host–virus interactions that may be exploited to modify ARV replication and selectivity (54). However, because ARV and MRV differ in host range, receptor usage, and viral proteins, these findings should not be directly extrapolated to pelareorep.

## 5 ReoV delivery strategies

Although intravenous infusion of oncolytic viruses is convenient, their high intrinsic immunogenicity triggers rapid clearance by the host immune system, which limits their effectiveness. In addition, systemically administered free virus has limited tumor specificity and is susceptible to nonspecific sequestration and immune clearance. This reduces therapeutic efficacy and raises the risk of off-target toxicity (55). To overcome these delivery barriers, researchers have developed diverse carrier strategies.

### 5.1 Delivery via mesenchymal stem cell vectors

Mesenchymal stem cells (MSCs) are increasingly utilized as biological delivery vehicles for oncolytic viruses due to their intrinsic “tumor-homing” properties. In a C57BL/6 mouse model, ReoV-loaded MSCs not only traversed physiological barriers to migrate to the tumor site but also significantly enhanced IFN-γ secretion, thereby effectively inhibiting tumor growth (56). To tackle the issue of viral clearance by neutralizing antibodies (NAbs) prior to reaching target cells, researchers incorporated reovirus (T3D strain) into adipose-derived mesenchymal stem cells (AD-MSCs). *In vitro* studies revealed that these engineered cells, while preserving their normal phenotype, can modify the antitumor microenvironment by upregulating pro-inflammatory factors such as TNF-α and nitric oxide (NO), while downregulating immunosuppressive factors including TGF-β1 and IL-10. Moreover, when co-cultured with TC-1 tumor cells, they effectively activated both intrinsic and extrinsic apoptotic pathways in the tumor cells (57). Additionally, human umbilical cord mesenchymal stem cells (hUC-MSCs) exhibited dual advantages in an acute myeloid leukemia (AML) model; they not only functioned as a “physical barrier” against antibody-mediated viral neutralization but also significantly improved the targeted delivery and oncolytic efficacy of ReoV by modulating signaling pathways within the microenvironment (58).

### 5.2 Delivery via extracellular vesicles

Small extracellular vesicles (sEVs) are emerging as potential biomimetic viral nanocarriers due to their excellent biocompatibility, low immunogenicity, and capacity to traverse biological barriers. A recent study demonstrated that sEVs derived from human AD-MSCs effectively encapsulated ReoV particles, resulting in a significant reduction of colorectal cancer cell viability, with live cell counts decreasing by 60.3% in the HCT116 cell line and 42.5% in the SW480 cell line. The primary advantage of this nanodelivery system is its ability to sustain potent oncolytic activity even in the presence of anti-σ1 neutralizing antibodies and immune serum. Furthermore, the system is rapidly internalized by cancer cells within 4 hours. Notably, it overcomes the reliance of ReoV infection on the JAM-A receptor and exhibits strong cytotoxic effects against drug-resistant colon cancer cells lacking the JAM-A gene (59). vesicles (Reo-SEV) secreted by ReoV-infected B16 melanoma cells also exhibit significant therapeutic potential. Reo-SEV efficiently induces tumor cell apoptosis *in vitro* and markedly inhibits the growth of subcutaneous tumors in *in vivo* models, while also promoting the recruitment of CD8+ T cells to tumor tissues. These findings suggest that Reo-SEV, by synergistically activating both innate and adaptive immune responses, may serve as a novel oncolytic agent with broad therapeutic prospects (60).

### 5.3 Cationic liposome delivery

Cationic liposomes, as a classic non-viral nanocarrier, have been instrumental in tackling the challenge of pre-existing immunity prevalent in clinical environments. Research has demonstrated that the liposome encapsulation strategy effectively protects ReoV from neutralization by high concentrations of neutralizing antibodies found in the ascites of ovarian cancer patients. This approach significantly restores and enhances ReoV’s targeted cytotoxic activity against ovarian cancer cells, thereby offering a novel clinical strategy to overcome barriers to intraperitoneal viral delivery (61).

### 5.4 Other delivery strategies

In addition to the previously mentioned carriers, researchers are actively investigating alternative delivery strategies. Early studies (62) involving cellular carriers demonstrated that a composite system of immature dendritic cells and lymphokine-activated killer cells effectively reduced antibody neutralization in ovarian cancer ascites while significantly enhancing *in vitro* oncolytic activity. In the realm of physical permeation enhancement, ultrasound-targeted microbubble disruption technology has been shown to mechanically improve viral penetration and diffusion within dense tumor stroma (63). Moreover, oral delivery—an emerging non-invasive administration route—can elicit a robust systemic antitumor immune response by exploiting the intricate cascading interactions between gut microbiota and gut-associated lymphoid tissue (GALT) (64). In the future, the organic integration of cellular carriers, biomimetic nanomaterials, and physical permeation enhancement technologies is anticipated to fundamentally address the immunological and anatomical barriers that impede the systemic administration of ReoV. This advancement in delivery technology is expected to markedly improve the clinical translation efficiency of reoviral therapy and expand its potential applications in the treatment of solid tumors.

## 6 Translational challenges of ReoV monotherapy and combination therapies

### 6.1 Limitations of studies on the antitumor effects of ReoV monotherapy

ReoV monotherapy has shown biological activity in preclinical and early clinical studies; however, their therapeutic efficacy is often limited by complex biological barriers. In breast cancer, for example, tumor-associated metalloproteinase-mediated viral degradation and the development of resistance in target cells significantly impede their direct oncolytic activity (65, 66). Moreover, recent studies suggest that (67) ReoV exhibits limited antitumor activity as a monotherapy *in vivo*; ReoV monotherapy may induce adaptive immune resistance, including compensatory PD-L1 upregulation. Safety concerns may also be greater in patients with underlying liver injury, although this issue requires further clinical evaluation. Nonetheless, ReoV’s distinct mechanistic advantages in reshaping the tumor microenvironment (TME) and reversing immunosuppression (68) indicate that these limitations provide a rationale for combination therapy foundation for combination therapy. Consequently, current research therefore increasingly focuses on combination regimens: by strategically combining ReoV with immune checkpoint inhibitors, such as anti-PD-L1 agents or other therapeutic modalities, the goal is to overcome the mechanisms underlying monotherapy resistance and ultimately achieve a synergistic antitumor effect.

### 6.2 Challenges in ReoV combination therapy research

#### 6.2.1 Combination with chemotherapeutic agents

Many chemotherapy agents, while effectively targeting proliferative tumor cells, can also alter the intracellular environment to facilitate viral replication, thereby producing synergistic oncolytic activity. Synergy with ReoV is primarily achieved by enhancing the tumor immune microenvironment or by directly activating the apoptotic pathway. In the context of ovarian cancer treatment, researchers have found (69) that the effects of chemotherapy–ReoV combinations are agent-dependent: its pairing with cisplatin, gemcitabine, or cyclophosphamide can yield therapeutic advantages by enhancing tumor-cell killing, reshaping antitumor immunity, or diminishing neutralizing antibody responses. Conversely, when combined with paclitaxel, the treatment inhibits viral replication by reversing the hypoxic microenvironment and did not improve antitumor efficacy. In a separate study concerning pancreatic ductal adenocarcinoma (PDAC) (70), pelareorep plus gemcitabine demonstrated a relatively manageable toxicity profile; however, it was limited by the inherent barriers posed by the dense matrix and immunosuppressive microenvironment of PDAC, the clearance of the virus by pre-existing neutralizing antibodies, and the reductions in chemotherapy dose intensity. In the treatment of triple-negative breast cancer, reovirus and doxorubicin exhibit synergistic potential; however, their clinical translation is significantly hindered by three primary factors. First, at the pharmacological level, challenges arise from the physical antagonism between drug-loading capacity and viral activity, along with unclear drug release kinetics. Second, at the biological level, barriers include variability in treatment efficacy due to tumor heterogeneity, incomplete activation of immune pathways, and poorly defined antimetastatic mechanisms. Third, at the clinical level, limitations stem from reliance on local administration, unknown systemic safety of engineered viral strains, and the absence of predictive biomarkers for patient stratification (71). To address these challenges, future research should prioritize elucidating the release mechanism, developing strategies for systemic administration, and evaluating systemic safety and efficacy in clinically relevant models.

#### 6.2.2 Combined treatment with radiotherapy

Early studies (72) have shown that the combination of ReoV and radiotherapy can yield a synergistic pro-apoptotic effect, irrespective of the administration sequence. However, the clinical translation of this combination therapy encounters several challenges. At the clinical application level, current safety conclusions are primarily based on intratumoral injections in palliative superficial tumors, making it difficult to directly extrapolate findings to deep-seated tumors or radical treatment scenarios, where prolonged drug administration may influence antibody pharmacokinetics (73). Mechanistically, although this therapy can activate the mitochondrial apoptosis pathway in certain melanoma subtypes, it demonstrates resistance in KRAS-mutant tumor models. Additionally, the inhibition of PKR that is independent of CUG2, along with the nuclear–cytoplasmic signaling network associated with the CUG2 pathway, remains inadequately understood (74). More critically, most prior studies have not evaluated this therapy in immunocompetent tumor models. As previously noted, while radiotherapy-induced viral replication is cell line-dependent and constitutes a fundamental aspect of its antitumor efficacy, the dose fractionation regimen and the dose-response relationship with specific immune responses require further investigation (75). Consequently, future research must urgently explore the immunostimulatory potential of this combination therapy within the realistic tumor-immune microenvironment, precisely calibrate the combined dosing regimen to mitigate antagonistic risks, and actively validate the systemic efficacy and targeted distribution of intravenous administration, with the ultimate aim of maximizing the synergistic benefits of ReoV and radiotherapy.

#### 6.2.3 Combination therapy with immune checkpoint inhibitors

Anti-PD-1 antibodies may enhance the antitumor efficacy of reovirus through complementary innate and adaptive immune mechanisms. On the one hand, they enhance NK-cell function while mitigating immune suppression mediated by regulatory T cells. On the other hand, they significantly promote CD8^+^ T cell-mediated Th1-type immunity and tumor antigen-specific responses (18). The combination of immunotherapy and reovirus presents certain limitations. In a study of microsatellite stable (MSS) colorectal cancer (76), this combination therapy enhanced antitumor activity in MSS colorectal cancer models. However, its clinical translation is hindered by a lack of synergistic effects in microsatellite unstable (MSI) colorectal cancer, suboptimal efficacy of monotherapy, and inherent challenges in virus-targeted delivery. Additionally, the immunomodulatory effects of this therapy vary significantly across different microsatellite states, exemplified by the downregulation of innate immune sensing in MSI and the reduced NK-cell abundance in MSS. These factors further restrict its broad clinical applicability. In a study investigating the mouse models of metastatic breast and ovarian cancer (77),the effectiveness of combining reovirus with natural killer T-cell immunotherapy was found to be heavily influenced by the type of tumor. While reovirus exhibited a synergistic effect in the ID8 model, its efficacy was compromised in the 4T1 model due to various mechanistic deficiencies such as limited direct cytotoxicity, limited ability to enhance antigen presentation, limited pro-inflammatory capabilities, and inadequate induction of immunogenic cell death. When used in conjunction with NKT cell immunotherapy, reovirus not only proved ineffective but also potentially impeded the efficacy of the NKT-cell therapy alone. In a study on prostate cancer (78), the effectiveness of combining oncolytic reovirus with PD-1 or CD73 antibodies was strongly schedule-dependent, wherein antibody efficacy appeared to depend on prior immune priming by reovirus. Moreover, the synergistic effects anticipated from anti-CD73 and anti-PD-1 treatments were not realized in the prostate cancer model, underscoring the limited additional benefits of this multi-target combination regimen within a specific immunosuppressive microenvironment. To overcome the aforementioned conversion bottlenecks, genetic engineering of reovirus and delivery strategies have become even more critical.

#### 6.2.4 Other combination therapy strategies

In the context of oncolytic virus combination therapy for colorectal cancer (CRC), existing studies have yielded promising preclinical evidence while also revealing critical limitations that require urgent attention. Optimized dosing and sequencing strategies are required for advancing this therapeutic approach. On the one hand, the combined use of reovirus and STING agonists (79) demonstrates systemic or distant antitumor activity in CRC mouse models. However, this strategy is hindered by potentially opposing effects on immune signaling; specifically, the virus not only antagonizes the STING-induced type I interferon response *in vitro* but also did not significantly increase CD8^+^ T-cell infiltration within the tumor microenvironment. Furthermore, the non-T-cell-mediated synergistic mechanisms remain poorly understood, and the efficacy of this approach is dependent on the STING status of the experimental model, which restricts its applicability in heterogeneous tumors. Future investigations should focus on sequential drug administration to mitigate early conflicts in signaling pathways, thoroughly examine the contributions of innate immune cells, and identify more universal biomarkers to inform clinical applications. On the other hand, the strategy that combines reovirus with the autophagy inducer carbamazepine significantly decreased the viability of KRAS-mutant CRC cells, demonstrating greater efficacy than monotherapy. Nevertheless, its activity was largely restricted to KRAS-mutant cells, along with the delayed induction of core autophagy-related genes, exacerbates the inherent limitations of oncolytic viruses within complex immunosuppressive microenvironments (80). In the future, meticulous patient stratification based on KRAS mutation status, coupled with the potential introduction of additional targeted or immunotherapeutic agents that can enhance microenvironment remodeling or initiate autophagy, may help to improve the efficacy of this two-agent combination (**Table 2**).

**Table 2:** Summary of clinical trials evaluating ReoV-based therapies.

Tumor type	Regimen	Phase	Recruitment status	N	Test Number
Relapsed and Refractory Plasma Cell Myeloma	Bortezomib and Dexamethasone	I	Completed	14	NCT02514382
Childhood Astrocytoma, Childhood Atypical Teratoid/Rhabdoid Tumor, Diffuse Intrinsic Pontine Glioma,etc.	Sargramostim	I	Completed	6	NCT02444546
Anemia, Recurrent Multiple Myeloma, Refractory Multiple Myeloma	Carfilzomib and Dexamethasone	I	Completed	12	NCT02101944
Recurrent Melanoma Stage IV Melanoma		II	Completed	21	NCT00651157
Unspecified Childhood Solid Tumor, Protocol Specific	Cyclophosphamide	I	Completed	26	NCT01240538
Metastatic Melanoma	Carboplatin/Paclitaxel	II	Completed	14	NCT00984464
Recurrent Fallopian Tube Carcinoma Recurrent Ovarian Carcinoma Recurrent Primary Peritoneal Carcinoma	Single drug	I	Completed	70	NCT00602277
Osteosarcoma Ewing Sarcoma Family Tumors Malignant Fibrous Histiocytoma,etc.	Single drug	II	Completed	53	NCT00503295
Metastatic or Recurrent Squamous Cell Carcinoma of the Lung	Paclitaxel Carboplatin	II	Completed	32	NCT00998192
KRAS Mutant Metastatic Colorectal Cancer	Irinotecan/Fluorouracil/Leucovorin (FOLFIRI), Bevacizumab	I	Completed	36	NCT01274624
Pancreatic Acinar Cell Carcinoma Pancreatic Ductal Adenocarcinoma Recurrent Pancreatic Carcinoma	Carboplatin/Paclitaxel	II	Completed	73	NCT01280058
Recurrent Fallopian Tube Carcinoma Recurrent Ovarian Carcinoma Recurrent Primary Peritoneal Carcinoma	Paclitaxel	II	Completed	108	NCT01199263
Carcinoma, Non-small Cell Lung	Carboplatin/Paclitaxel	II	Completed	37	NCT00861627
Breast Cancer Metastatic	Paclitaxel/Avelumab	II	Active, not recruiting	48	NCT04215146
Recurrent Plasma Cell MyelomaRefractory Plasma Cell Myeloma	Bortezomib Dexamethasone Pembrolizumab	I/II	Active, not recruiting	10	NCT05514990

Data were obtained from ClinicalTrials.gov.

## 7 Challenges and future perspectives

ReoV has emerged as a promising cancer immunotherapy platform because of its dual mechanism of direct oncolysis and immune activation. However, several barriers continue to restrict its clinical translation. First, the clinical safety and off-target risks must be closely monitored. While ReoV-based therapies generally exhibit a controllable safety profile, treatment-related adverse events—such as fever, transient lymphocytopenia, and systemic inflammatory responses—cannot be overlooked, particularly in patients with compromised immune systems, necessitating a more cautious safety assessment (70). Second, technical barriers in viral genetic engineering hinder the development of the next generation of oncolytic viruses. Although the genetic modification of reovirus to overcome the limitations of natural receptors is a necessary trend in clinical development, efficient reverse genetics tools for highly active strains remain limited. Additionally, the insertion of exogenous targeted peptides, such as the RGD sequence, frequently results in an unbalanced trade-off among the virus’s structural stability, oncolytic titer, and targeting safety. This constitutes a major technical bottleneck in this field. Third, systemic delivery barriers and host immune responses significantly impede therapeutic efficacy. Neutralizing antibodies present in the circulatory system, rapid clearance by the reticuloendothelial system, and limited intratumoral penetration collectively obstruct the effective systemic delivery of free viruses. This lack of targeting, coupled with the absence of protective circulatory mechanisms, frequently results in clinical dose limitations, thereby posing a substantial challenge to the development of novel delivery vectors (81). Furthermore, tumor heterogeneity and variability among patients greatly complicate clinical translation. High levels of heterogeneity render “one-size-fits-all” treatment regimens ineffective, creating considerable obstacles for clinical trial design and endpoint assessment. Scientific research must prioritize systematically addressing these challenges (82).

Future advancements in ReoV therapy will rely heavily on synergistic progress across several dimensions: establishing a more comprehensive safety monitoring and intervention system; developing a new generation of engineered strains with enhanced genomic stability; validating and optimizing systemic delivery platforms capable of evading immune clearance, such as nanomaterials or cellular carriers; and implementing biomarker-guided, precise patient stratification. Ultimately, the core objective driving reoviral therapy toward clinical application will be to translate this potent immune-activating mechanism into tangible and sustained clinical survival benefits (**Figure 3**).

**Figure 3:**
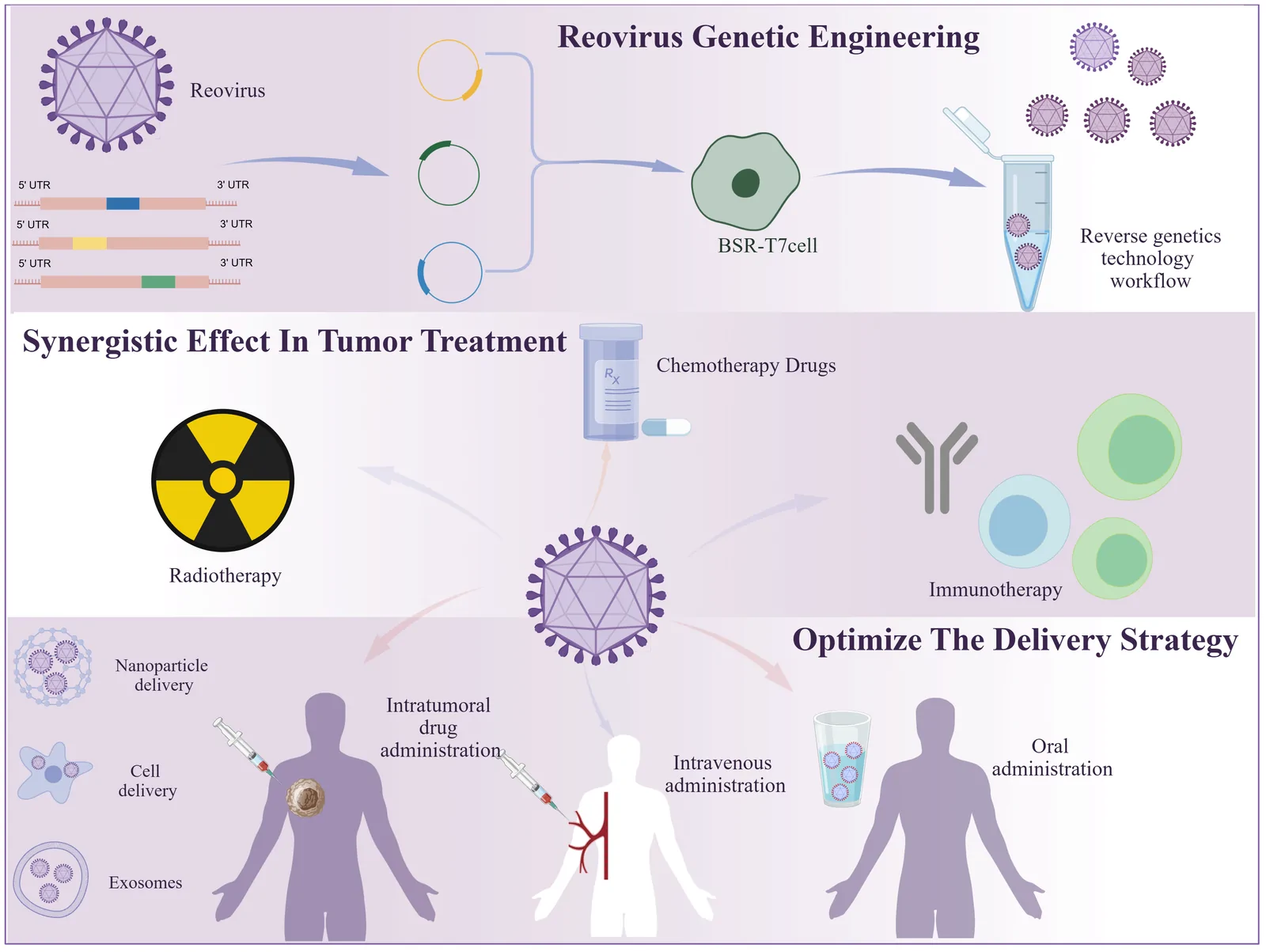
Overview of reovirus engineering, combination therapy, and delivery strategies. Reverse-genetics approaches enable capsid modification and transgene insertion. Reovirus may be combined with radiotherapy, chemotherapy, immunotherapy, and targeted therapies and administered through intratumoral, intravenous, or oral routes. Nanoparticles, cellular carriers, and extracellular vesicles represent investigational delivery platforms.
